# How are individual-level social capital and poverty associated with health equity? A study from two Chinese cities

**DOI:** 10.1186/1475-9276-8-2

**Published:** 2009-02-15

**Authors:** Xiaojie Sun, Clas Rehnberg, Qingyue Meng

**Affiliations:** 1 Center for Health Management and Policy, Shandong University, Jinan 250012, PR China; 2 Medical Management Center, Karolinska Institutet, SE-171 77 Stockholm, Sweden

## Abstract

**Background:**

A growing body of literature has demonstrated that higher social capital is associated with improved health conditions. However, some research indicated that the association between social capital and health was substantially attenuated after adjustment for material deprivation. Studies exploring the association between poverty, social capital and health still have some serious limitations. In China, health equity studies focusing on urban poor are scarce. The purpose of this study is therefore to examine how poverty and individual-level social capital in urban China are associated with health equity.

**Methods:**

Our study is based on a household study sample consisting of 1605 participants in two Chinese cities. For all participants, data on personal characteristics, health status, health care utilisation and social capital were collected. Factor analysis was performed to extract social capital factors. Dichotomised social capital factors were used for logistic regression models. A synergy index (if it is above 1, we can know the existence of the co-operative effect) was computed to examine the interaction effect between lack of social capital and poverty.

**Results:**

Results indicated the poor had an obviously higher probability of belonging to the low individual-level social capital group in all the five dimensions, with the adjusted odds ratios ranging from 1.42 to 2.12. When the other variables were controlled for in the total sample, neighbourhood cohesion (NC), and reciprocity and social support (RSS) were statistically associated with poor self-rated health (NC: OR = 1.40; RSS: OR = 1.34). However, for the non-poor sub-sample, no social capital variable was a statistically significant predictor. The synergy index between low individual-level NC and poverty, and between low individual-level RSS and poverty were 1.22 and 1.28, respectively, indicating an aggravating effect between them.

**Conclusion:**

In this study, we have shown that the interaction effect between poverty and lack of social capital (NC and RSS) was a good predictor of poor SRH in urban China. Improving NC and RSS may be helpful in reducing health inequity; however, poverty reduction is more important and therefore should be implemented at the same time. Policies that attempt to improve health equity via social capital, but neglect poverty intervention, would be counter-productive.

## Background

Social capital, which is a key term in sociology, is often used in economics and other fields as well. An extensive literature has accumulated on the relationship between social capital and economic development [1]. Economists from the World Bank reported that social capital, which is characterised by trust and social bond, played an important role in poverty reduction. Different groups could support each other and supply health or education services. In addition, it has been noted that the poor could benefit more from group memberships and active participation in decision making than the rich[2]. This could be explained by the rich's ability to buy health or education services irrespective of their social capital.

Could social capital reduce health inequity that is linked to poverty in a similar manner as in poverty reduction? Different scholars have proposed different explanations to account for the existence of health inequity. *The neo-materialists *emphasise that even in the most affluent societies the poor could still suffer major material deprivations that directly cause their health to deteriorate. However, the proponents of *psychosocial mechanisms *stress that the perception of living in an unequal society could be so corrosive of social relationships that this can have tangible consequences on the health of the population. Still, others argue that material and psychosocial interpretations are not mutually exclusive; nor is it usually possible to disentangle their effects from one another[3]. A growing body of evidence has demonstrated that higher social capital is associated with improved health conditions [4-8]. Some researchers have suggested three possible mechanisms to explain the improvement of health outcomes: (1) influence health-related behaviours, (2) influence access to services and amenities and (3) affect psychosocial processes[9]. However, the relationships between income inequality, social capital and health are still disputed. Presently, there are two ways of understanding the phrase "income inequality and health": one is the individual-level associations between income and health, the other is the ecological-level associations between a measure of income inequality (such as the Gini coefficient) and aggregate health (e.g., mortality rates). At the ecological level (state/country), Kawachi et al. [7] have reported that income inequality leads to increased mortality via disinvestment in social capital. At the individual level, the association between income, social capital and health has also been the explored. Stafford et al[10] found that social capital was highly correlated with material deprivation and that all associations between social capital and health were attenuated after adjustment for material deprivation. Based on a qualitative study, Cattell observed that, although social capital could buffer its harsher effects, the concept was inadequate in explaining the deleterious effects of poverty on health and well being[11]. Although few studies have tested this hypothesis, it seems that there are interactive effects between lack of social capital and poverty. In addition, many studies on social capital and health have two other limitations. First, poor people or those within deviant or marginalised communities has been excluded[12]. Second, social capital is a multifaceted concept and thus more indicators should be included in addition to group membership and feelings of trust. Therefore, a study that includes both poor and non-poor people and that uses multi-dimension social capital indicators would be more effective in revealing the relationships between poverty, social capital and health.

Despite the enthusiasm of sociologists, economists and epidemiologists, social capital remains under-theorised with no unified definition. Bourdieu regarded social capital as "the aggregate of the actual or potential resources which are linked to possession of a durable network"[13]. Coleman argued that "social capital inheres in the structure of the relations between persons and among persons" [14]. These two views are typical for a "network view" of social capital. Some economists have regarded individual social capital as "a person's social characteristics which enables him to reap market and non-market returns from interactions with others"[15]. This position is similar to the network view. Putnam consider social capital as "features of social organisation, such as trust, norms, and networks, that can improve the efficiency of society by facilitating co-ordinated actions" [16]. His "communitarian view" of social capital emphasises social cohesion. Some epidemiologists have defined social capital as features of social structures (interpersonal trust, norms of reciprocity and mutual aid) that act as resources for individuals and facilitate collective action[7,9]. Hence, these epidemiologists also placed emphasis on "social cohesion", but considered it as a resource.

In a Chinese context, "investments" in social capital to develop and maintain social networks may provide individuals with access to resources and supports. We argue that an operational definition of "network view" may be more suitable. Recently, the Policy Research Initiative (PRI) of Canada proposed a definition[17] based on social networks as its central component. That is, "social capital refers to the networks of social relations that may provide individuals and groups with access to resources and supports." We use this as our operational definition, where we argue that resources also include features of social structures (interpersonal trust, norms of reciprocity and mutual aid).

Since the implementation of the reforming and opening policy in the late 1970s, China has made significant progress in improving the living standards of the people of China. However, health development does not automatically follow economic growth. On the one hand, during the transition from planning economy to market economy, the income gaps between urban and rural areas, the rich and the poor and the eastern and western regions have widened; on the other, the gaps in health and health care between these different population groups are also widening ([18,19]. The increasing gaps in health and health care are ascribed to income gaps, changes of health care financing and organisations, low health insurance coverage and relaxed public health [18,20]. Linlin Hu[21] found that the income inequality was closely related to health inequality; however, income inequality alone could not fully explain health inequality. Liangshu Qi [22] noted that the correlation between income and health was not obvious in rural areas, whereas in urban areas the higher the income, the better the health. Shaojin Wang[23] argued that the "income inequality-health" approach[24] is applicable and adaptable to China. Wang further pointed out some possible mechanisms, including the impact of income inequality on social capital.

Most studies analysing the relationship between social capital and health have been implemented in high-income countries (e.g., the USA, the UK, Australia, Canada and Sweden), whereas few studies have been carried out in middle- and low-income countries (e.g., China). Social capital is a highly context- and culturally bound conception, which can differ in its manifestation and meaning across cultures/countries. During the transition in China from a planned economy to market economy, the resource allocation mechanism has changed where the administration power has weakened and the market-dominant means have not been completely established. Thus, social capital has become an important approach in establishing and maintaining a trust relationship, exchanging benefits with each other and seeking support[25]. Traditional social capital based on ancestral relationships and extended family networks dominates in China, whereas macro-level modern social capital based on extensive trust and co-operation is still lacking. To our knowledge, only a few studies have looked at the issue of how poverty and social capital are associated with health equity in China. Winnie Yip et al. explored the association between social capital and health in rural China[26]; however, the authors concluded, "major differences in socioeconomic conditions between the urban and rural areas warrant separate analyses". In fact, urban poor are rapidly increasing in recent years though health equity studies focusing on urban poor are sparse. According to the Ministry of Civil Affairs of China, at the end of 2003, 30 million urban residents lived below the absolute poverty line and between 30 and 40% suffered from various diseases [27]. The China-UK Urban Health and Poverty Project (UHPP) was a medical assistance programme that implemented in Xining and Yinchuan, China. The aim of the programme was to help China construct a sustainable and replicable community health system that provides improved access for the urban poor. Data from the UHPP survey included both the poor and the non-poor living in the same community. Using these survey-data, we pose the following questions: (1) What is the association between poverty and individual-level social capital in Western China (more than a half of urban poor live there)? (2) At the individual level (and after controlling for other variables), which statistical effects will social capital dimensions have on SRH? (3) Is there an interaction effect between poverty and social capital on SRH?

## Methods and data

### Study setting

Our study is based on a household survey from July–August 2006 with cross-sectional data from two capital cities of Western China (Xining City of Qinghai Province and Yinchuan City of the Ningxia Hui Autonomous Region). Qinghai and Ningxia are both undeveloped areas in Northwest China. The disposable income per capita of urban residents was 8472 yuan (1130 US$) in 2003 for the whole country; however, for Xining it was only 7025 yuan (936.7 US$) by the end of 2003 and for Yinchuan 7245 yuan (966 US$) by the end of 2004. The population of urban residents in Xining was about one million, 5.27% of whom were poor people receiving a minimum living allowance. Of these poor people, 91.9% were not covered by the urban health insurance scheme (by the end of 2003). The population in Yinchuan was about 760,000, of whom 5.47% were classified as poor. Of these, 92% were not covered by the urban health insurance scheme (by the end of 2004).

In 1999, the minimum living standard security system was established in most cities of China. Urban residents whose average family income is lower than the minimum living standard can apply for the minimum living allowance. Investigation of the family's income should be conducted before issuance of the minimum living allowance, the level of which is calculated as the difference between the family per-capita income and the minimum living standard. By the middle of 2006, the minimum living standard was 165 yuan (22 US$) and 180 yuan (24 US$) per capita per month in Xining and Yinchuan, respectively.

### Sampling and data collection

There are four districts in Xining and three in Yinchuan. All seven districts were included in this survey. In Xining, 14 communities from 7 sub-districts were selected according to geographical distribution and population density. We selected poor households based on the lists of households enjoying the minimum living allowance supplied by the local communities. In each community, 25 poor households were selected using systematic sampling. The same size of non-poor households was randomly selected in the same community. In Yinchuan, 31 communities from 18 sub-districts were selected. In each community, 13 poor households and 13 non-poor households were selected according to the same rules outlined above. The final sample included 801 poor households and 804 non-poor households. The total population of selected poor households accounted for 4.38% and 5.30% of the total urban poor in Xining and Yinchuan, respectively. In each family, one family member (aged 15 years and over) who was living at home at the time of the present survey received a closed questionnaire about personal and family characteristics, health status, health care utilisation and social capital. Of the 1605 individuals investigated, 1509 (or 94%) answered all the social capital questions. The response rates for the survey among the poor, among the non-poor and overall were 93.38%, 94.65% and 94.02%, respectively. 74% of the valid questionnaires were answered by the householder, 21.7% by the spouse of the householder and the others by the child or parent of the householder.

### Measures and definitions

#### Health status

Self-rated health (SRH) has proved to be a more robust predictor of mortality than "objective" measures of health status or social-economic status (SES)[28,29]. Accordingly, in this study the primary outcome measure used is SRH in response to the question: "How would you describe your present health status? Would you say it is good, fair, or poor?" For the analysis, we categorised the answers "good" and "fair" as "not poor" (SRH: Y = 0) and "poor" as "poor" (SRH: Y = 1).

#### Individual characteristics

Key demographic variables (age, gender, ethnicity and marital status), socioeconomic variables (educational level, family economic status and logarithm of the family monthly income), one geographical variable (residence district) and health status (with or without chronic illness) are included. Family economic status is measured by a dichotomous variable (poverty or non-poverty). Family monthly income was estimated by the responders. The residence districts included four districts from Xining and three from Yinchuan.

#### Social capital

Many health studies[7,30-32] are constrained in that they use only secondary data and are unable to design their own measures of social capital. Some efforts have been made to achieve a sufficiently comprehensive measure[32,33]. However, the social capital measures in this study were only a small part of the household survey and thus did not allow a long social capital questionnaire. Based on the operational definition, reviewing other comprehensive measure instruments[33,34], we selected some commonly used indicators and adapted them to the Chinese context. Table 1 shows these indicators in relation to dimensions of reciprocity and social support (RSS), social participation, perception of trust and safety, interpersonal relationship network and neighbourhood cohesion (NC). RSS indicators involved support received from relatives, friends and neighbours when for example confined to bed by illness or in trouble. For the dimension "social participation", the indicators included the quantity and frequency of group participation and the frequency of community participation. To measure "perception of trust and safety", we used such indicators as trust in community residents, asking neighbours to watch your home and perception of community safety. Interpersonal relationship network was measured by the number of close relatives, the number of close friends and the occupations of these persons (such occupations as officials, teachers and lawyers usually control important resources). The responder was given an option to choose if his/her close relatives and friends were fitted into the following occupations. The categories were government official, head of enterprises or institutions, doctor, teacher, lawyer and business leaders. However, as this was a multi-choice question, a corresponding quantitative number was given to show that the question which had multiple answers. For example, if the responder selected doctor, teacher and lawyer, "three" was assigned to the corresponding quantitative variable. NC indicators were mainly about the daily communications within neighbourhoods.

**Table 1:** Social capital dimensions and related indicators

**Dimensions**	**Indicators**
**Reciprocity and social support**	Do you believe that when you are ill or feel uncomfortable someone will care for you?
	Do you believe that if you are ill your neighbours will help you?
	Do you believe that if you have private problems close friends or relatives will discuss them with you?
**Social participation**	In how many civic/political/cultural/religious groups or organisations have you participated?
	Do you often take part in activities held by these groups or organisations?
	How many times have you participated in community collective activities within the past year?
**Perception of trust and safety**	Do you believe that the majority of residents in your community can be trusted?
	Would you like to ask your neighbours to watch your home when you are away?
	How do you feel about public safety in your community?
	Do you believe that most community residents will participate in a programme conducted by the community that benefits only a few residents?
**Interpersonal relationship network**	How many close relatives do you have?
	How many close friends do you have?
	Which of the following occupations are your close relatives and friends pursuing: government official, head of enterprises or institutions, doctor, teacher, lawyer and business leader?
**Neighbourhood cohesion**	Do you often visit your neighbours?
	Do you often invite your neighbours into your home?
	Do you often chat with your neighbours?

### Statistical analyses

The statistical analysis included three stages: (1) factor analysis was employed to extract social capital dimensions; (2) logistic regression models were conducted to explore the association between poverty and social capital, social capital and health and poverty and health; and (3) synergy index was computed to examine the interaction effect between lack of social capital and poverty as a predictor of poor self-rated health (SRH).

#### Factor analysis

After missing cases were deleted, 1509 respondents were included in the factor analysis. Five factors were extracted with eigenvalues above 1.0. After running a varimax orthogonal rotation, the five factors explained 59.4% of the total variance. Table 2 shows the factor loadings of all the social capital indicators. Except for the factors "perception of trust and safety" (alpha = 0.37) and "interpersonal relationship network" (alpha = 0.58), the internal consistency reliability of the other factors was relatively good (Table 3).

**Table 2:** factor loadings of social capital indicators

	**Main Components**				
**Variables**	**1**	**2**	**3**	**4**	**5**
1. Do you believe that when you are ill or feel uncomfortable someone will care for you?	.051	.067	**.776**	.123	.137
2. Do you believe that if you are ill your neighbours will help you?	.303	.010	**.663**	.062	.197
3. Do you believe that if you have private problems close friends or relatives will discuss them with you?	.097	.079	**.783**	.162	.072
4. In how many civic/political/cultural/religious groups or organisations have you participated?	.015	**.838**	.138	.035	-.046
5. Do you often take part in activities held by these groups or organisations?	.060	**.855**	.145	.158	.047
6. How many times have you participated in community collective activities within the past year?	.137	**.655**	-.142	.167	.224
7. Do you believe that the majority of residents in your community can be trusted?	.199	.049	-.029	.089	.**736**
8. Would you like to ask your neighbours to watch your home when you are away?	**.466**	.004	.048	-.038	.398
9. How do you feel about public safety in your community?	-.029	.022	.249	-.094	**.511**
10. Do you believe that most community residents will participate in a programme conducted by the community that benefits only a few residents?	.049	.085	.145	.078	**.613**
11. How many close relatives do you have?	.092	.001	.044	**.806**	.039
12. How many close friends do you have?	.098	.119	.178	**.753**	.012
13. Which of the following occupations are your close relatives and friends pursuing: government official, head of enterprises or institutions, doctor, teacher, lawyer and business leader?	.016	.196	.091	**.647**	.015
14. Do you often visit your neighbours?	**.711**	.031	.090	.084	.171
15. Do you often invite your neighbours into your home?	**.868**	.116	.143	.092	.020
16. Do you often chat with your neighbours?	**.856**	.068	.122	.088	-.020

**Table 3:** Selected results related to factor analysis

**Extracted dimension**	**Eigenvalue**	**Internal consistency reliability (Cronbach's α)**
**Neighbourhood cohesion**	3.80	0.68
**Social participation**	1.84	0.67
**Reciprocity and social support**	1.44	0.71
**Interpersonal relationship network**	1.34	**0.58**
**Perception of trust and safety**	1.07	**0.37**

When compared with the original dimensions (Table 1), the results of the factor analysis were in perfect accordance with these dimensions, with the exception that the indicator: "when you are not at home, would you like to ask your neighbours to watch your home?" was mainly explained by "NC", and not by "trust and perception of safety".

The factor score of each dimension was dichotomised into a binary variable, and the mean of factor score was used as the switch point: high individual-level social capital (factor score ≥ 0) and low individual-level social capital (factor score < 0). Using the dichotomised social capital variables, Spearman correlation coefficients were computed between the social capital factors (Table 4). At the individual level, the highest correlation coefficient was between NC and social participation (0.084, p < 0.05). These results show that different social capital factors in this study were relatively independent.

**Table 4:** Spearman correlation coefficients between social capital factors

	**Individual level**				
	**Factor1**	**Factor2**	**Factor3**	**Factor4**	**Factor5**
**Individual level**					
					
**Factor1**	1.000	0.084**	0.031	0.069**	-0.001
**Factor2**	0.084**	1.000	0.027	0.050	-0.002
**Factor3**	0.031	0.027	1.000	0.007	-0.005
**Factor4**	0.069**	0.050	0.007	1.000	0.016
**Factor5**	-0.001	-0.002	-0.005	0.016	1.000

**P < 0.01.

factor1 = neighborhood cohesion, factor2 = social participation, factor3 = reciprocity and social support, factor4 = interpersonal relationship network and factor5 = perception of trust and safety.

#### Logistic regression

To compare the differences of social capital between the poor and non-poor, we computed adjusted odds ratios, using logistic regression models to control for such variables as gender, age, ethnicity, marital status, education level and residence district.

Then logistic regression models were used to explore the predictors of poor SRH.

The following basic model was used, where Y is poor SRH and X is the influencing factor.

(1) $$\text{ln}\frac{\mathrm{Pr}(Y=1)}{\mathrm{Pr}(Y=0)}=\ln(\text{odds})=\beta_{0}+\beta_{1}X_{1}+\cdots +\beta_{p}X_{p}$$

Based on this basic model, six logistic regression models were constructed: independent variables used in model 1A included demographic variables and individual-level social capital variables. The study participants of model 1A were the total sample. Based on model 1A, model 1B introduced the variable economic status (poverty or non-poverty). Model 2A and 3A used the same independent variables as model 1A, but the participants were the poor and non-poor sub-samples. Model 2B and 3B introduced the variable log (family monthly income) in order to explore the impact of income within the poor and the non-poor groups.

#### Method for interaction effects

To quantify interaction based on Rothman's[35] model, the synergy index (S) was computed using the following equation:

(2) $$\text{S}=\frac{RR_{11}-1}{\left(RR_{10}-1)+(RR_{01}-1\right)}$$

RR (relative risk) denotes the incidence rate ratios with RR_00 _(unexposed to poverty and lack of social capital) as a denominator. RR_10 _and RR_01 _are the rate ratios for those with one exposure but without the other exposure, whereas RR_11 _is the rate ratios for those with both exposures. The index denotes synergy (the phenomenon in which two discrete influences or agents acting together create an effect greater than that predicted by knowing only the separate effects of the individual agents) if its value exceeds 1.0 and antagonism (the phenomenon where two agents in combination have an overall effect that is less than that predicted from their individual effects) if its value is less than 1.0. We used the method proposed by Jun Zhang[36] to obtain a corrected RR based on adjusted OR. That is:

(3) $$\text{RR}=\frac{OR}{\left(1-P_{00})+(P_{00}\times OR\right)}$$

In this study, P_00 _denoted the percent of poor SRH in the non-exposed group (non-poverty and high social capital). Adjusted OR was computed using a logistic regression model. Except for the two variables ("economic status" and the significant social capital variable in the total sample) whose interaction effect was analysed, the other studied variables were controlled for.

Fitting of the logistic regression model was tested with the maximum likelihood method. The Enter method was used to introduce variables. Adjusting for all other variables in the regression equation, OR with 95% confidence intervals (CIs) and significance levels were calculated on the binary response (poor SRH) by changes in the predicting variable. All data were analysed with the SPSS 11.5 (SPSS, Inc, Chicago, IL) statistical software package.

### Ethical clearance

In this study, consent forms were used to get approvals from participants on the collection and use of the data. Interviewees in this study were informed about the purposes and objectives of this study and accepted to participate. Agreement was reached between researchers and study participants on the use of the data for scientific purposes.

This study was supported by the China-UK UHPP. Ethical clearance was obtained from the Senior Management Committee of the UHPP.

## Results

### Descriptive statistics

Table 5 summarises the descriptive statistics of the poor and non-poor groups. The proportions of males in the poor and non-poor groups were both nearly 40%. About one fourth of the sample was aged 65 years and over (25.3% of the poor, 25.6% of the non-poor). The ethnicity of the majority was Han, and the percent of poor Hui people was 10.4% higher than that of non-poor Hui people (χ2 = 25.93, p < 0.001). Nearly one half (46.8%) of the poor were illiterate or had only a primary school education, whereas 42.7% of the non-poor had a high school education or higher. The majority of non-poor (83.7%) were first married, whereas the percent of divorced in the poor was 6.4% (χ2 = 27.81, p < 0.001) higher than those in the non-poor and the percent of widowed in the poor was 18.77% (χ2 = 83.61, p < 0.001) higher than those in the non-poor. The family monthly income of non-poor households was 2.87 times as much as that of poor households. The prevalence of chronic illness among the poor was 13.8% higher than that of the non-poor (χ2 = 28.62, p < 0.001). The percent of poor SRH of the poor was more than two times that of the non-poor.

**Table 5:** Descriptive statistics

**Indicators**	**Poor (N = 748)**	**Non-poor (N = 761)**
**Independent variables**		
**Gender (%)**		
Male	39.6	42.2
Female	59.9	57.7
Missing	0.5	0.1
**Age, years (%)**		
15–44	36.5	33.5
45–64	37.7	40.0
65-	25.3	25.6
Missing	0.5	0.9
**Ethnicity (%)**		
Han	73.7	84.0
Hui	24.9	14.5
Others	1.5	1.6
**Education level (%)**		
Illiterate	25.0	10.1
Primary school	21.8	14.5
Middle school	34.2	32.1
High school	13.9	26.0
Technical secondary school	3.3	5.9
Junior college	1.5	8.2
University and above	0.1	2.6
Missing	0.1	0.7
**Marital status (%)**		
Never married	3.7	1.7
First married	55.6	83.7
Remarried	2.4	1.3
Divorced	9.0	2.6
Widowed	29.3	10.5
Missing	0.0	0.1
**Chronic illness (%)**		
With chronic illness	48.4	34.6
Missing	0.0	0.3
Family monthly income* (yuan)	441.0	1267.0
**Dependent variable (%)**		
Poor SRH	38.2	17.2
Missing	0.9	1.8

*Means

### The association between poverty and low individual-level social capital

The association between poverty and low individual-level social capital is given in Table 6. The poor showed a higher percent of low individual-level social capital than the non-poor in all five dimensions, with the ratios of poor to non-poor ranging from 1.16 to 1.39. After controlling for other variables (gender, age, ethnicity, marital status, education level and residence district), compared with the non-poor group, the poor group had an obviously higher probability of belonging to the low individual-level social capital group in each dimension, with the adjusted odds ratios ranging from 1.42 (the dimension: NC) to 2.12 (the dimension: interpersonal relationship network).

**Table 6:** Comparison of individual-level social capital

Indicators	Poor (N = 999)	Non-poor (N = 977)	Poor/non-poor	Adjusted Odds Ratio* (reference: non-poor group)
**Low individual-level social capital**				
Neighbourhood cohesion	55.2	44.8	1.23	1.66** (1.36–2.03)
Social participation	67.4	58.0	1.16	1.64** (1.30–2.06)
Reciprocity and social support	54.5	44.8	1.22	1.42** (1.16–1.75)
Interpersonal relationship network	71.8	51.6	1.39	2.12** (1.71–2.64)
Perception of trust and safety	48.7	40.9	1.19	1.45**(1.18–1.77)

**P < 0.01.

Here, other variables (gender, age, ethnicity, marital status, education level and residence district), were controlled for by logistic regression models.

### Logistic regression models

Table 7 presents the factors that influence poor SRH. In model 1A (the total sample), higher education level was associated with a lower probability of poor SRH. People who resided in the Chengzhong district of Xining had the lowest probability of poor SRH (OR = 0.50). Chronic illness was closely associated with poor SRH (OR = 12.48). Among the five individual-level social capital variables, only NC and RSS were statistically significant predictors, the odds ratios were respectively 1.45 and 1.36. After "economic status" was controlled for in model 1B, where the probability of poor SRH in poor people was 2.17 times higher than in non-poor people, small changes were seen. The odds ratios of two age groups (45–64 and 65-) increased from 1.18 and 0.90 to 1.39 and 1.19, respectively. The odds ratio of each education level group also increased to a small extent. However, the odds ratios of NC and RSS dropped by 0.05 and 0.02, respectively.

**Table 7:** Logistic models explaining poor SRH by the total sample and the sub-samples of the poor and non-poor (odds ratio, 95%CI)

**Dependent variables**	**Poor SRH (total sample)**		**Poor SRH (poor)**		**Poor SRH (non-poor)**	
	**Model 1A**	**Model 1B**	**Model 2A**	**Model 2B**	**Model 3A**	**Model 3B**
**Independent variables**						
**Constant**	0.08**	0.05**	0.05**	0.48	0.27	3.2
**Gender (ref: male)**	1.21 (0.92–1.59)	1.25 (0.95–1.66)	1.12 (0.78–1.60)	1.14 (0.79–1.64)	1.71 * (1.06–2.75)	1.70 * (1.05–2.75)
**Age, years** **(ref:15–44)**						
45–64	1.18 (0.85–1.63)	1.39 * (1.00–1.94)	2.05 ** (1.37–3.07)	2.02 ** (1.34–3.04)	0.52 * (0.28–0.98)	0.57 (0.30–1.07)
65-	0.90 (0.60–1.35)	1.19 (0.78–1.80)	1.30 (0.74–2.25)	1.34 (0.76–2.34)	0.62 (0.30–1.25)	0.67 (0.33–1.39)
**Ethnicity (ref: Han)**						
Hui	1.30 (0.92–1.84)	1.22 (0.85–1.73)	1.23 (0.80–1.90)	1.11 (0.71–1.74)	1.02 (0.53–1.98)	0.99 (0.51–1.92)
Others	0.50 (0.17–1.52)	0.51 (0.17–1.56)	0.79 (0.20–3.22)	0.67 (0.16–2.88)	0.11 (0.01–1.17)	0.13 (0.01–1.36)
**Education level (ref: illiterate)**						
Primary school	0.62 * (0.42–0.93)	0.67 (0.45–1.01)	0.86 (0.53–1.41)	0.91 (0.55–1.51)	0.31 ** (0.14–0.67)	0.32 ** (0.15–0.70)
Middle school	0.50 ** (0.34–0.75)	0.57 ** (0.38–0.87)	0.69 (0.41–1.15)	0.74 (0.44–1.25)	0.34 ** (0.16–0.72)	0.35 ** (0.17–0.75)
High school	0.46 ** (0.29–0.74)	0.57 * (0.35–0.92)	0.94 (0.50–1.76)	0.98 (0.51–1.85)	0.21 ** (0.09–0.49)	0.21 ** (0.09–0.50)
Technical secondary school	0.22 ** (0.10–0.49)	0.27 ** (0.12–0.59)	0.36 (0.12–1.07)	0.40 (0.13–1.21)	0.12 ** (0.03–0.42)	0.13 ** (0.04–0.48)
Junior college and above	0.29 ** (0.14–0.61)	0.38 * (0.18–0.82)	0.59 (0.19–1.90)	0.72 (0.22–2.32)	0.22 ** (0.07–0.69)	0.27 * (0.09–0.87)
**Marriage status (ref: never married)**						
First married	1.13 (0.60–2.13)	1.27 (0.68–2.40)	1.77 (0.83–3.80)	2.01 (0.92–4.39)	0.55 (0.16–1.94)	0.54 (0.15–1.93)
Remarried	0.82 (0.28–2.43)	0.90 (0.30–2.67)	2.05 (0.57–7.37)	2.12 (0.58–7.81)	0.07 * (0.01–0.82)	0.09 (0.01–1.09)
Divorced	1.72 (0.76–3.90)	1.62 (0.72–3.66)	2.23 (0.88–5.69)	2.27 (0.87–5.87)	0.68 (0.09–4.82)	0.55 (0.08–3.94)
Widowed	1.61 (0.79–3.29)	1.45 (0.71–2.96)	2.54 * (1.07–6.02)	2.38 (0.99–5.71)	0.57 (0.13–2.40)	0.43 (0.10–1.90)
**Residence district (ref: Chengxi)**						
Chengbei	0.65 (0.40–1.07)	0.63 (0.38–1.05)	0.76 (0.39–1.49)	0.91 (0.46–1.80)	0.43 * (0.20–0.96)	0.47 (0.21–1.03)
Chengdong	1.22 (0.72–2.05)	1.17 (0.69–1.99)	1.79 (0.92–3.52)	2.01* (1.01–4.01)	0.53 (0.21–1.35)	0.59 (0.23–1.51)
Chengzhong	0.50** (0.30–0.84)	0.47 ** (0.28–0.80)	0.71 (0.36–1.41)	0.74 (0.37–1.46)	0.21 ** (0.09–0.52)	0.22 ** (0.09–0.53)
Jinfeng	0.75 (0.46–1.23)	0.65 (0.39–1.08)	1.11 (0.58–2.11)	1.27 (0.65–2.47)	0.25 ** (0.10–0.61)	0.28 ** (0.11–0.69)
Xixia	0.91 (0.55–1.51)	0.80 (0.48–1.34)	1.13 (0.58–2.21)	1.30 (0.65–2.58)	0.40 * (0.17–0.93)	0.43 (0.18–1.02)
Xingqing	0.85 (0.55–1.31)	0.80 (0.51–1.24)	1.09 (0.61–1.97)	1.16 (0.64–2.10)	0.41 * (0.20–0.86)	0.45 * (0.22–0.95)
**Economic status (ref: non-poverty)**	-	2.17 ** (1.64–2.88)	-	-	-	-
**Log (family monthly income)**	-	-	-	0.39** (0.20–0.74)	**-**	0.43 (0.19–1.02)
**Chronic illness (ref: no chronic illness)**	12.49** (9.49–16.44)	11.84** (8.98–15.60)	9.07** (6.45–12.75)	9.35** (6.61–13.22)	28.57** (16.36–49.89)	27.80** (15.90–48.63)
**Neighbourhood cohesion (ref: high level)**	1.45** (1.12–1.88)	1.40* (1.08–1.82)	1.42* (1.01–1.98)	1.40 (1.00–1.98)	1.26 (0.81–1.98)	1.23 (0.78–1.93)
**Social participation (ref: high level)**	0.85 (0.64–1.15)	0.78 (0.58–1.06)	0.69 (0.46–1.04)	0.69 (0.46–1.05)	0.97 (0.60–1.56)	0.89 (0.55–1.45)
**Reciprocity and social support (ref: high level)**	1.36* (1.05–1.76)	1.34* (1.03–1.74)	1.30 (0.93–1.81)	1.31 (0.94–1.83)	1.35 (0.86–2.12)	1.29 (0.82–2.03)
**Interpersonal relationship network (ref: high level)**	1.16 (0.88–1.53)	1.04 (0.78–1.39)	1.22 (0.84–1.78)	1.16 (0.79–1.70)	0.67 (0.42–1.09)	0.66 (0.41–1.07)
**Perception of trust and safety (ref: high level)**	1.00 (0.78–1.30)	0.98 (0.75–1.26)	0.91 (0.65–1.26)	0.90 (0.64–1.26)	1.00 (0.63–1.57)	1.00 (0.63–1.58)
**N**	1919	1919	976	957	943	939
**χ2 (df)**	606.24(26)	636.13(27)	308.41(26)	312.23(27)	292.86(26)	293.36(27)
**-2 log likelihood**	1595.37	1565.48	958.35	930.72	553.13	547.95
**Percentage correct of predicted values**	79.4	80.5	75.7	76.2	86.2	86.4

*P < 0.05, **P < 0.01.

Model 1A was based on the total sample. Model 1B introduced economic status (poor or non-poor). Model 2A and 3A were based on two sub-samples: the poor and the non-poor, using the same variables as model 1A. Model 2B and 3B introduced an income variable (log (family monthly income)).

In model 2A (the poor sub-sample), both of the "45–64" and "65-" groups had higher probabilities of poor SRH. The widowed group had the highest probability of poor SRH (OR = 2.54). Among the five individual-level social capital variables, only NC was a significant predictor of poor SRH (OR = 1.42). In model 2B, with an increase in Log (family monthly income), the probability of poor SRH showed a descending trend (OR = 0.39). Gender, education level, ethnicity, marriage status, residence district were not significant predictors before and after introducing "Log (family monthly income)".

In model 3A (the non-poor sub-sample), females had a higher probability of poor SRH than the males (OR = 1.71). None of the social capital variables revealed statistical significance. The variable "Log (family monthly income)" was introduced into model 3B, and with an increase in Log (family monthly income), the probability of poor SRH showed a descending trend (OR = 0.43). Age group, ethnicity, and marriage status were not significant predictors before and after introducing "Log (family monthly income)".

Table 8 presents the interaction effects between lack of social capital and poverty. The results indicated that if exposed only to low individual-level NC or poverty, the adjusted OR of poor SRH is 1.28 and 2.09, respectively; however, if exposed to low individual-level NC and poverty simultaneously, the adjusted OR increased markedly to 2.88. The synergy index between low individual-level NC and poverty was as high as 1.22, suggesting an interaction effect between them. If exposed only to low individual-level RSS or poverty, the adjusted OR of poor SRH is 1.30 and 2.03, respectively; however, if exposed to low individual-level RSS and poverty at the same time, the adjusted OR increased markedly to 2.99. The synergy index between low individual-level RSS and poverty was 1.28, indicating an interaction between them.

**Table 8:** Evaluation of interaction effects between lack of social capital and poverty on poor SRH (adjusted odds ratios)

***Indicators***	neighbourhood cohesion (NC)		reciprocity and social support (RSS)	
	
	**high**	**low**	**high**	**low**
**Non-poverty**	1	1.28(0.85–1.91)	1	1.30 (0.87–1.95)
**Poverty**	2.09 (1.42–3.08)	2.88(1.96–4.24)	2.03(1.37–3.02)	2.99 (2.04–4.39)

**P**_00_	15.1%		16.8%	

***Synergy index***	1.22		1.28	

Except for the two variables whose interaction effect was analysed, the other variables (gender, age, ethnicity, education level, marriage status, residence district, with or without chronic illness, and the other four social capital variables) were controlled for using logistic regression models.

## Discussion

Our analyses has shown that after controlling for other variables the poor had higher probabilities of belonging to the low individual-level social capital group in all the five dimensions (RSS, social participation, perception of trust and safety, interpersonal relationship network and NC) examined in this paper. For the total sample, high individual-level NC and RSS were statistically associated with a lower probability of poor SRH. However, for the non-poor sub-sample, no social capital variable was a statistically significant predictor. The synergy index indicated that there was an interaction effect between low individual-level NC and poverty, and between low individual-level RSS and poverty.

### Poverty and social capital

The positive association between poverty and lack of social capital has been discussed extensively[7,37]. After controlling for other variables in the present study, the poor had higher probabilities of belonging to the low individual-level social capital group in all the five dimensions. This finding indicates that lack of social capital is closely associated with poverty. One apparent conclusion is that poverty will lead to a decline of social capital, which has two possible explanations: first, material or non-material investments are necessary because a member of any network (formal or informal) is expected to contribute with something; however, the poor may not have enough material resources to offer and their non-material resources might not be appreciated by those from a different background[38]. This plight will result in the shrinking of social networks of poor people. Second, people with lower income tend to participate less and to be less trusting than people with higher levels of income[37]. Yet, low social capital may also reduce the ability to buffer the impact of poverty. For most poor people, their social networks could do little to help them get rid of the poverty. Moreover, the poor usually receive very limited financial and physical support from their social networks. As a result, they get caught in a vicious spiral. In an effort to break this spiral of poverty, the World Bank began to promote the increased social capital formation as a major strategy for poverty reduction.

### Social capital and poor SRH

The association between social support and health has been analysed elsewhere, so we will focus on NC in this paper. In several studies, a positive effect of NC or neighbourhood social capital on health has been reported [10,39,40]. The Chinese saying, "*a neighbour that is near is better than a brother far off*", nicely illustrates the importance of NC in traditional Chinese culture. However, with the acceleration of urbanisation, more and more people from different places and work units reside together, which have caused some urban residents in China "*not to communicate with strange neighbours*". People having lived together for a long time in the same community may nevertheless not know each other, let alone help one another. This tendency is particularly held for vulnerable groups (the poor, the old and the handicapped) in neighbourhoods with less cohesion. In such neighbourhoods, few people would be willing to take care of these vulnerable people (e.g., lending them money or taking them to hospital for treatment of a medical condition). At the neighbourhood level in China, there are several explanations as to how social capital could affect individual health. One explanation is that social capital could influence health behaviours by promoting rapid diffusion of health information[41], or by exerting social control over deviant health-related behaviours [9]. A second explanation is that, in a cohesive neighbourhood, people will have higher levels of access to health services. A third explanation pertains to the notion that social capital could influence an individual's health via psychosocial processes by providing affective support and acting as a source of self-esteem and mutual respect [42].

In our study, social participation was not statistically associated with SRH at the individual level. This finding is consistent with a recent study from rural China [26]. There are several accounts for this lack of association. First, whether social participation is positive, negative, or meaningless to SRH is still unclear [43-46]. Second, for some unknown reason, few people in China have participated in formal groups or social activities (political or non-political). Rather, Chinese people seem to prefer to take part in informal groups or activities. Consequently, our measures of social participation may have underestimated this latter type of social participation. Furthermore, there were no evidence in our study that the interpersonal relationship network was statistically associated with SRH. Similarly, Pevalin [39] showed that contact with friends did not reduce the likelihood of common mental illness and poor SRH. Different people have different relationship networks: some networks may be good for health, whereas some may be neutral, or even cause harm. An unclear result may appear because the positive and negative effects from interpersonal relationship networks may counteract one another.

Contrary to other studies[5,47-49], our observations suggest that the perception of trust and safety was not statistically associated with poor SRH. One explanation for the contradictory results is the relatively low reliability of the "Perception of trust and safety" dimension. In addition, we only measured "trust in people from the respondents' communities", which may underestimate the impact of trust. However, in Veenstra's study [44], measurement of trust included trust in government, neighbours, people from the respondents' communities and people in general: even here trust was found not to be associated with SRH.

### The interaction effects between social capital and poverty on health

In this study, only in the poor subsample, lack of social capital (NC) was statistically associated with poor SRH. Similarly, a recent study done by Stafford and his colleague also showed that, for people living in deprived circumstances only, associations between neighbourhood social capital and common mental disorders (CMD) were seen[50]. Multiple, overlapping forms of discrimination or marginalisation (such as being female, lower education and unemployment) may magnify the effects of poverty on health [51]. Lack of social capital can be regarded as one form of marginalisation. Our results of the interaction effect between lack of social capital and poverty support this argument. This observation is helpful in explaining the finding that all of the associations between social capital and health were attenuated after adjustment for material deprivation. We agree with Carlson's [52] contention that, although both the economic factors and some aspects of social capital played a role in the area differences in SRH, economic factors appeared to be more important. As Collier [53] (pp. 19–41) noted, "*the poor have a lower opportunity cost of time and a lower stock of financial and physical capital than the rich*"; thus, they may "*choose to rely more upon social capital than the better off*" because "*social interaction is time-intensive*" and "*social capital can often substitute for private capital*". This notion may help to explain the interaction between lack of social capital and poverty. It can therefore be inferred that improving the NC and RSS of the poor may be helpful in reducing health inequity. However, because of the spiral effect of social capital and poverty, this relief effect would be only transitory if poverty was not simultaneously reduced.

### Limitations

We want to pay attention to six main limitations in our study. One limitation is that our analysis was cross-sectional and hence the data did not permit causal inferences between lack of social capital and poverty and lack of social capital and poor SRH. For example, we could not conclude whether poverty results in the decline of social capital, or whether the lack of social capital produces poverty. A longitudinal cohort study would be needed to answer this question. The second limitation is that only a small proportion of households were selected in each community. Further, only those who were at home when we visited answered such subjective questions as SRH and social capital indicators, which could introduce selection and reporting bias to this study. For a micro-level social capital study, a sample with more representative respondents from the same community would help to eliminate this limitation. Also, because of this limitation, the contextual effect could not be analyzed in this study, which should be further explored in the future by increasing samples from each community and some community-level variables. The third limitation concerns the aggregation of social capital variables. Because of the characteristics of the data, we did not aggregate individual-level social capital into community-level or higher levels. The fourth limitation is that if the implementation of UHPP has changed the health status and social capital of local poor people, it would have been better to select a sample without any special intervening programme. Another limitation is that the internal consistency reliability of two of our dimensions ("perception of trust and safety" and "interpersonal relationship network") was relatively low, which may underestimate the effects of these dimensions. The final limitation refers to the possibility that the results may not be generalisable to all urban Chinese because there are many cultural and socioeconomic disparities among different Chinese cities.

Despite these limitations, our study still provides some valuable insights into the complex association between poverty, social capital and health in urban China.

## Conclusion

In this study, we have shown that the interaction effect between poverty and lack of social capital (NC and RSS) was a good predictor of poor SRH in urban China. Improving NC and RSS may be helpful in reducing health inequity; however, poverty reduction is more important and therefore should be implemented at the same time. Policies that attempt to improve health equity via social capital, but neglect poverty intervention, would be counter-productive.

## Competing interests

The authors declare that they have no competing interests.

## Authors' contributions

XS participated in conceiving and implementing the study and had the main responsibility for writing the manuscript. CR is the co-supervisor of XS, who directed data analysis and manuscript writing. QM is the main supervisor of XS, who directed the conceiving and implementing of this study and manuscript writing.
